# Caspase-2 Mediated Apoptotic and Necrotic Murine Macrophage Cell Death Induced by Rough *Brucella abortus*

**DOI:** 10.1371/journal.pone.0006830

**Published:** 2009-08-28

**Authors:** Fang Chen, Yongqun He

**Affiliations:** Unit for Laboratory Animal Medicine and Department of Microbiology and Immunology, University of Michigan Medical School, Ann Arbor, Michigan, United States of America; University of Birmingham, United Kingdom

## Abstract

*Brucella* species are Gram-negative, facultative intracellular bacteria that cause zoonotic brucellosis. Survival and replication inside macrophages is critical for establishment of chronic *Brucella* infection. Virulent smooth *B. abortus* strain 2308 inhibits programmed macrophage cell death and replicates inside macrophages. Cattle *B. abortus* vaccine strain RB51 is an attenuated rough, lipopolysaccharide O antigen-deficient mutant derived from smooth strain 2308. *B. abortus* rough mutant RA1 contains a single *wboA* gene mutation in strain 2308. Our studies demonstrated that live RB51 and RA1, but not strain 2308 or heat-killed *Brucella*, induced both apoptotic and necrotic cell death in murine RAW264.7 macrophages and bone marrow derived macrophages. The same phenomenon was also observed in primary mouse peritoneal macrophages from mice immunized intraperitoneally with vaccine strain RB51 using the same dose as regularly performed in protection studies. Programmed macrophage cell death induced by RB51 and RA1 was inhibited by a caspase-2 inhibitor (Z-VDVAD-FMK). Caspase-2 enzyme activation and cleavage were observed at the early infection stage in macrophages infected with RB51 and RA1 but not strain 2308. The inhibition of macrophage cell death promoted the survival of rough *Brucella* cells inside macrophages. The critical role of caspase-2 in mediating rough *B. abortus* induced macrophage cell death was confirmed using caspase-2 specific shRNA. The mitochondrial apoptosis pathway was activated in macrophages infected with rough *B. abortus* as demonstrated by increase in mitochondrial membrane permeability and the release of cytochrome *c* to cytoplasm in macrophages infected with rough *Brucella*. These results demonstrate that rough *B. abortus* strains RB51 and RA1 induce apoptotic and necrotic murine macrophage cell death that is mediated by caspase-2. The biological relevance of *Brucella* O antigen and caspase-2-mediated macrophage cell death in *Brucella* pathogenesis and protective *Brucella* immunity is discussed.

## Introduction

*Brucella* species are Gram-negative, facultative intracellular bacteria that cause brucellosis in humans and animals [1]. Human brucellosis remains the most common zoonotic disease worldwide with more than 500,000 new cases annually [2]. *B. abortus* infects primarily cattle and is one of the common *Brucella* species that afflict humans [2]. The brucellae disseminate or spread via the blood and lymphatics where they multiply inside phagocytes and cause bacteremia. Unlike many pathogenic bacteria, *Brucella* lacks most of the classical virulence factors such as invasive proteases, exotoxins, capsules, fimbriae, virulence plasmids, and lysogenic phages [3]. Instead, *Brucella* virulence relies on its ability to survive and replicate in the vacuolar phagocytic compartments of macrophages. *Brucella* lipopolysaccharide (LPS) is a *Brucella* virulence factor [4]. The LPS has three domains: lipid A, the core oligosaccharide, and the O antigen (O-polysaccharide, or O-side chain). The brucellae exhibit two phenotypes, i.e., smooth and rough. Smooth *Brucella* strains include a complete LPS, while rough *Brucella* strains do not contain or produce very low level O-antigen. Smooth virulent *Brucella* strains, e.g., *B. abortus* strain 2308 (S2308), inhibit programmed cell death of infected human and mouse macrophages [5], [6], [7]. For example, smooth *B. suis* infection inhibits spontaneously occurring apoptosis in human macrophages [5], [6], [7]. The inhibition of cell death facilitates the survival and replication of smooth *Brucella* strains inside macrophages. In contrast, many rough derivatives of *B. abortus*, *B. melitensis*, and *B. suis*, deficient in the LPS O antigen (O-side chain), can not survive inside macrophages and are therefore attenuated [8], [9]. A number of *B. abortus* rough strains are cytotoxic to macrophages and induce macrophage cell death [9], [10], [11], [12], [13]. The underlying details of the molecular mechanism of macrophage death induced by rough *Brucella* strains remain obscure.

Several types of programmed cell death have been defined. These include apoptosis, autophagy, and pyroptosis [14], [15], [16], [17]. These categories are based on criteria such as morphological alterations, initiation of a death signal, and the involvement of caspases. The most thoroughly studied aspect of programmed cell death is apoptosis. Apoptosis is mediated by a family of evolutionarily conserved proteases known as caspases [18]. Activation of caspases leads to cell shrinkage, nuclear condensation, and oligonucleosomal DNA fragmentation. There are two types of apoptotic caspases: initiator (apical) caspases and effector (executioner) caspases. Initiator caspases (e.g., caspase-2, 8, 9, 10) activate effector caspases (e.g., caspase-3, 6, 7) that digest specific proteins and/or activate other specific caspases (e.g., caspase-1, 4, 5, 11, 12, 13, 14). Two well-studied pathways of apoptosis are the mitochondrial (intrinsic) cell death pathway and the cell surface death receptor (extrinsic) pathway [19]. The mitochondrial cell death pathway is characterized by increased mitochondrial membrane permeability and the release of cytochrome *c* [20]. Necrosis refers to the sum of changes that have occurred secondary to cell death (e.g., apoptosis) after dead cells have reached equilibrium with their surroundings. According to recent nomenclature recommendations, necrosis is the end product of a cell death process instead of a form of cell death in itself [17], [21], [22].

Although macrophages are highly adept at destroying bacteria, modulation of macrophage cell death by some species of bacteria is emerging as an important pathogenesis mechanism. One such mechanism, pyroptosis, is a caspase-1-dependent pro-inflammatory cell death [17]. Caspase-1 is required for induction of macrophage cell death infected by many microbial species including *Salmonella typhimurium* [23], [24], [25], *Shigella flexneri* [26], *Legionella pneumophila* [27], and *Mycobacterium tuberculosis* [28]. The participating of caspase-2 has also been reported in *Salmonella*-infected macrophages [29]. Our previous microarray study indicates smooth virulent *B. melitensis* strain 16 M down-regulates caspase-2 transcriptional levels and inhibits transcription of genes involving mitochondria activities at an early stage of infection [6]. No caspases were up-regulated in strain 16M-infected macrophages. These results suggest that smooth virulent *Brucella* modulate macrophage cell death by inhibiting mitochondrial cell death pathway and caspase activation. The caspase gene expression profiles in macrophages infected with rough *Brucella* strains have not been studied.

We hypothesized that rough *B. abortus* strains would induce macrophage cell death through a caspase-dependent pathway. To test this hypothesis, we used rough *B. abortus* strains RB51 [30] and RA1 [31] as compared with their parent smooth virulent strain 2308 (S2308). The live attenuated rough strain RB51 serves as the official *Brucella* cattle vaccine and has been used in the US since 1996 [32]. The use of RB51 as a vaccine has the advantage that it does not stimulate *Brucella* O antigen specific antibodies and thus does not interfere with the diagnosis of wild type *Brucella* infection in the field. The rough phenotype of RB51 is due to the mutation of *wboA* that encodes a glycosyltransferase [33]. Compared to its parent strain S2308, RB51 has at least one additional mutation of *rpoB* [34]. *B. abortus* RA1 is a single *wboA* Tn5 rough mutant of S2308 [31]. Just as to RB51, RA1 lacks the O side chain and is attenuated. Elucidation of the mechanisms underlying of *Brucella* rough strains RB51 and RA1 induced macrophage response will improve our understanding of *Brucella* vaccine immunity and foster our goal of developing an effective vaccine against human brucellosis. The results presented here demonstrate that RB51 and RA1 induce a novel apoptotic and necrotic macrophage cell death pathway mediated by caspase-2.

## Results

### RB51 and RA1 induce apoptotic and necrotic macrophage cell death

**Figure 1:**
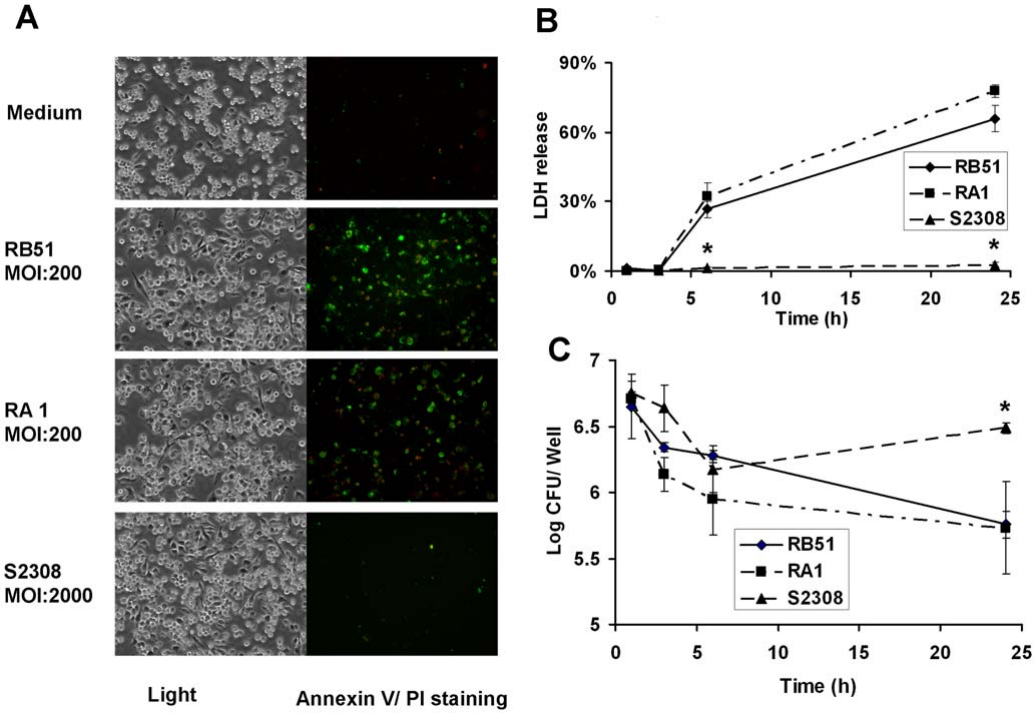
Cell death of macrophages infected with *B. abortus* strains RB51,RA1 and their parent wild type S2308. (A) RB51 and RA1 induced 61.5%±8.7% and 74.7%±5.6% macrophage cells death, respectively, at a MOI of 200 at 24 h post infection. *B. abortus* strain 2308 induced limited macrophage cell death even at the MOI of 2,000. (B) LDH release from RB51, RA1 and S2308 infected macrophages. The data represent the means±standard deviations for three independent experiments. The asterisk (*) represents the significant differences (*P*<0.05) of the LDH release level from macrophages infected by smooth S2308 compared to that from macrophages infected with rough strain RB51 or RA1. (C) Growth kinetics of RB51, RA1 and S2308 in macrophages. The number of internalized cells of S2308 at MOI 2,000 found was similar to the number of cells of internalized RB51 and RA1 at a MOI 200 at 1 h post infection. The asterisk (*) represents the significant differences (*P*<0.05) in survival level of smooth S2308 compared to that of rough strain RB51 or RA1 inside infected macrophages.

The programmed cell death of *Brucella*-infected macrophages was first analyzed by Annexin V (green) and propidium iodide (PI, red) staining. Fluorescein-conjugated Annexin V detects translocation of phosphatidylserine from the inner cell membrane to the outer cell membrane of cells at the early stage of apoptosis. PI stains the DNA of necrotic cells and/or cells at late stage of apoptosis [35]. The results of fluorescence staining of infected macrophages indicated that rough *B. abortus* strains RB51 and RA1 induce both apoptotic (stained green) and necrotic (stained green and red) macrophage cell death. At 24 h post infection with RB51 or RA1, 61.5%±8.7% or 74.7%±5.6% of the infected macrophages, respectively, underwent either apoptotic or necrotic cell death (Figure 1A, Table 1). The cytopathic effect of rough *Brucella* on macrophages was further confirmed by the lactate dehydrogenase (LDH) release assay, a nonradioactive cytotoxicity assay used to monitor the LDH release from dying membrane-damaged macrophages (Figure 1B).

**Table 1:** Kinetic analysis of *Brucella*-induced macrophage cell death.

MOI	H.P.I. a	S2308^b^	RB51^b^	HK-RB51^b^
2,000	4	−	++	-
	8	−	+++	-
	24	−	++++	+
	48	−	++++	+
200	4	−	+	-
	8	−	++	-
	24	−	+++	±
	48	−	++++	±
20	4	−	−	−
	8	−	±	−
	24	−	+	−
	48	−	+	−

a Hours Post Infection.

b Results of Annexin V and/or PI staining of macrophages infected with indicated bacteria: ++++, 75 to 100% positive; +++, 50 to 75% positive; ++, 25 to 50% positive; +, 5–25% positive; ±, <5% positive; -, no positive staining.

The kinetic profiles of intracellular survival of different *Brucella* strains were analyzed in parallel with the cell death of infected macrophages (Figure 1C). Macrophage cells were infected with RB51 or RA1 at a multiplicity of infection (MOI) of 200 that has been commonly used for *Brucella* infection studies [12], [36]. Neither RB51 nor RA1 survived inside macrophages as demonstrated by continuous decline of survived bacterial numbers inside macrophages. In contrast to these rough *Brucella* strains, a less amount of smooth strain 2308 (S2308) cells was taken up by macrophages at a MOI of 200 (data not shown). This observation is consistent with other reports [8], [12]. At 1 h post infection, the number of internalized S2308 cells found inside macrophages at the MOI of 2,000 was similar to the number of internalized RB51 or RA1 cells at a MOI 200 (Figure 1C). At 24 h post infection, the number of living S2308 cells was significantly higher than that of S2308 at 6 h post infection and that of RB51 or RA1 at 24 h post infection. These results indicated that S2308 survived and replicates inside macrophages. However, S2308 failed to induce any macrophage cell death, even with a MOI of 2,000, at 24 h post infection (Figure 1A and 1B).

**Figure 2:**
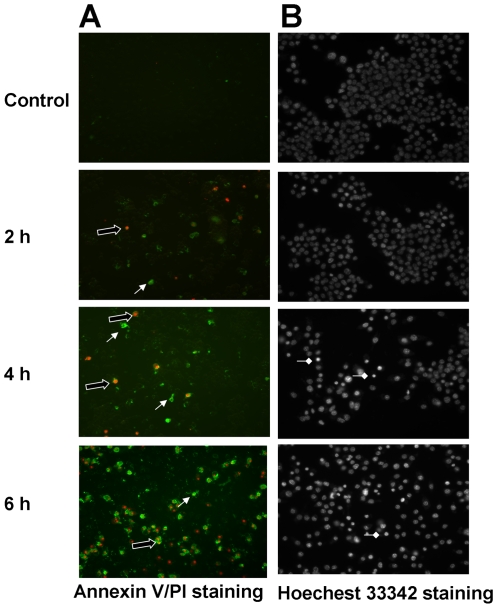
Apoptotic and necrotic macrophage cell death induced by RB51. (A) Annexin V/PI staining shows both apoptosis (solid arrow) and necrosis (hollow arrow) of macrophages at as early as 2 h post infection. (B) Staining with Hoechst 33342 shows cell shrinkage and nuclear condensation (diamond head arrow) of infected macrophages at 6 h post infection at a MOI of 200. The results are a representative of three independent experiments.

Programmed cell death of *Brucella*-infected macrophages was confirmed and further analyzed by Hoechst 33342 and Annexin V straining (Figure 2). Apoptotic cells were detectable as early as 2 h post infection with RB51 (Figure 2). The level of cell death increased to about 16.3%±2.1 at 4 h post infection. Approximately, 19.5%±2.9% macrophage cells exhibited both Annexin-V and PI positive (necrotic cell) among the dead cells population at 4 h post infection. At 6 h after infection, 26.6%±3.5% of cells was membrane-damaged macrophages as established by a lactate dehydrogenase (LDH) releasing test (Figure 1B). Of the dying cells, 70.3%±5.9% were necrotic or late-apoptotic cells. Others are early apoptotic cells (Figure 2A). RB51-induced apoptotic macrophage cell death was confirmed by staining with Hoechst 33342. The RB51- and RA1-infected macrophages exhibited typical signs of apoptosis, i.e., condensed nuclei and shrunken cells (Figure 2B).

**Figure 3:**
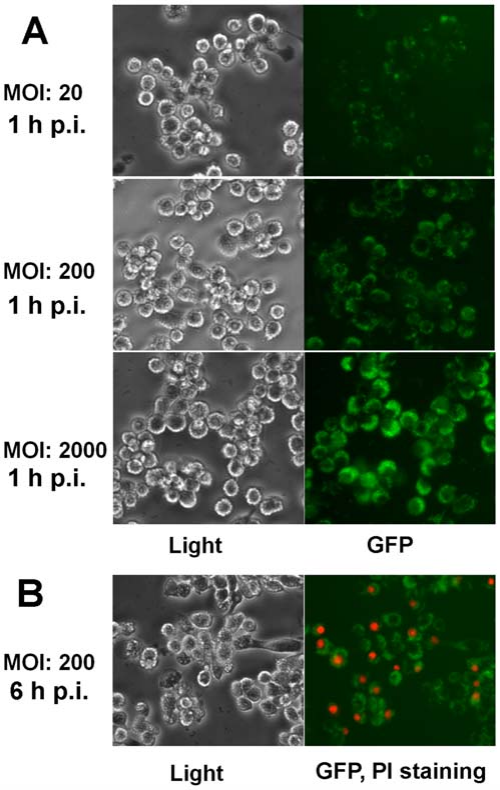
Analysis of the relationship between internalization of rough *Brucella* and macrophage cell death. (A) Uneven macrophage internalization of RB51GFP with different infection MOIs observed at 1 h post infection. (B) Most cell death occurred in macrophages infected with RB51GFP (MOI: 200) at 6 h post infection. The results are a representative of three independent experiments.

To specifically study *Brucella* internalization by individual macrophages, a recombinant strain RB51GFP that expresses the green fluorescent protein (GFP) [37] was used to infect macrophages. Over 95% of macrophages were infected with RB51GFP at any tested MOI (20, 200, and 2000) at 1 h post infection (Figure 3A). The bacteria infection rate for individual macrophages was uneven in any tested MOI. Some macrophages were infected with more bacteria than others. With a low MOI of 20, the majority of RB51GFP-infected macrophages contained 2–12 green fluorescence dots per macrophage (Figure 3A). The higher MOIs usually resulted in increased numbers of infected bacteria per macrophages. The PI staining of RB51GFP-infected macrophages allowed us to simultaneously monitor macrophage cell death and *Brucella* numbers inside macrophages (Figure 3B). It was found that most cell death occurred in *Brucella*-infected macrophages. For example, in macrophages infected with RB51GFP for 6 h (MOI: 200), most dying macrophages contained intracellular bacteria shown by green fluorescence (Figure 3B). A small number of PI-positive dying cells were not surrounded by green fluorescence probably due to the disruption of cell membrane leading to the release of bacteria. This possibility was observed by comparing the images of light microscopy and fluorescence microscopy.

To further determine the relationship between the infection dose of *Brucella* and macrophage cell death, a kinetic experiment was conducted (Table 1). In this experiment, macrophages were infected with different MOIs and cell death evaluated at different time points (4, 8, 24, and 48 h) following infection. It was found that the level and speed of macrophage cell death were dependent on the infection doses of rough *Brucella*. The macrophage cell death induced by RB51 with the MOI of 2,000 developed much faster than that with a MOI of 200 or 20 (Table 1). Heat killed RB51 (HK-RB51) only caused limited cell death (∼10%) at a MOI of 2000 at 24 and 48 h post infection. S2308 did not induce obvious cell death at any tested MOI.

**Figure 4:**
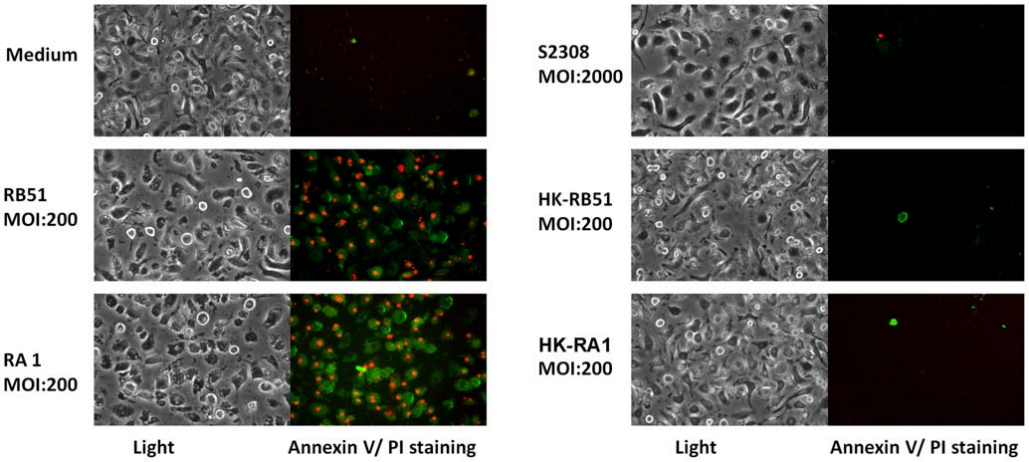
Apoptotic and necrotic cell death of bone marrow derived macrophages infected with viable rough *B. abortus* strains RB51 or RA1 (MOI: 200) at 24 h post infection. Smooth strain S2308 (MOI: 2000) and heat-killed RB51 and RA1 (equivalent MOI: 200) did not induce obvious macrophage cell death. The results are a representative of three independent experiments.

To test if the same cell death also occurs in bone marrow derived macrophages (BMDM) infected with rough *Brucella* strains, BMDM were prepared using BALB/c mice and infected with different *Brucella* strains. Live RB51 or RA1 was also capable of inducing apoptotic and necrotic cell death in BMDM. Heat-killed RB51 or RA1 did not cause obvious cell death with a MOI of 200. It suggests that viable rough strains are required to induce macrophage death. Live smooth strain S2308 did not induce cell death of infected BMDM (Figure 4).

### RB51 induces apoptosis and necrosis in primary peritoneal macrophages isolated from RB51-vaccinated mice

**Figure 5:**
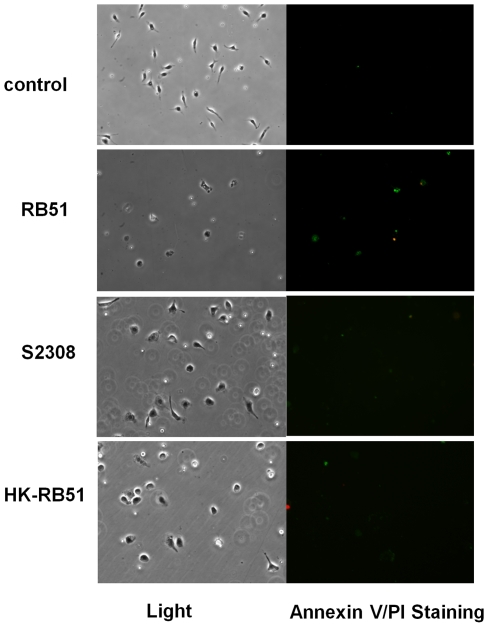
Apoptotic and necrotic cell death in peritoneal primary macrophages isolated from RB51-vaccinated mice. Annexin V/PI staining shows both apoptosis and necrosis of peritoneal macrophages at 24 h after infection with RB51. No obvious cell death was observed in peritoneal macrophages from mice injected (i.p.) with S2308 or heat-killed RB51 (HK-RB51). The results are a representative of three independent experiments.

Vaccine strain RB51 has been widely used for studying protective mechanisms against brucellosis using mice [30], [38]. In order to determine if RB51 or S2308 induces similar cell death in primary macrophages in RB51-infected mice, BALB/c mice were injected intraperitoneally (i.p.) with RB51, HK-RB51 or S2308 at a dose of 5×10^8^ CFU. This is the same dosage and injection route used for other typical RB51 mouse studies [39], [40]. Peritoneal macrophages were collected and monitored for induction of apoptosis and necrosis. In total, 38.4%±4.0% of the peritoneal macrophages from RB51-vaccinated mice showed apoptotic or necrotic staining at 24 h after infection (Figure 5). Macrophages infected with *Brucella* S2308 appeared to be activated as demonstrated by the larger size and irregular shape with long pseudopodia, as compared with noninfected macrophages that showed smaller size and oval shape with less short cytoplasmic processes. Similar observation was found in macrophages treated with HK-RB51. In addition, no obvious cell death was observed in macrophages from control mice injected with saline, HK-RB51 or S2308. These results indicate that RB51 induces apoptosis and necrosis in primary macrophages from RB51-vaccinated mice.

### Inhibition of caspase-2 inhibits RB51 and RA1-induced macrophage cell death

**Figure 6:**
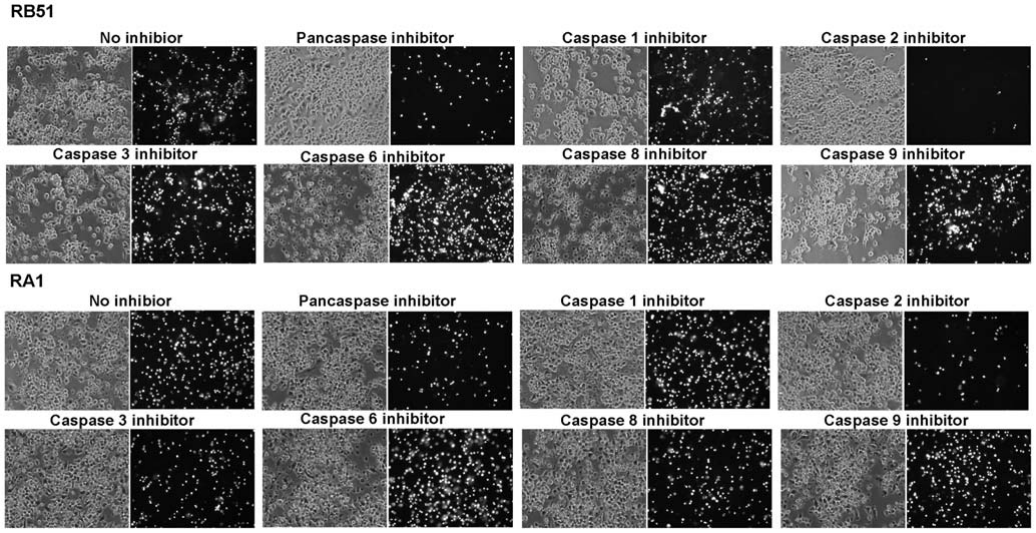
Inhibition of rough *B. abortus*-induced macrophage cell death by a pan-caspase inhibitor and a caspase-2 inhibitor. Macrophages were pretreated with individual caspase inhibitor and then infected with RB51 or RA1 at a MOI of 200. No inhibition was observed with any of the other caspase inhibitor. The results are a representative of three independent experiments.

The role of caspases in rough *B. abortus*-induced macrophage cell death was examined. Peptide-based substrates and inhibitors have been used extensively to monitor the association of caspases with cell death and to identify the caspases involved [41], [42]. Pretreatment of macrophages with the pan-caspase inhibitor Z-VAD-FMK prior to infection with RB51 or RA1 resulted in an approximate 69.4%±4.9% or 63.2%±4.8%, respectively, decrease in macrophage cell death at 24 h post infection compared to untreated infected macrophages (Figure 6). These results suggest that RB51 and RA1-induced macrophage cell death is mediated by a caspase(s). To determine the potential role of individual caspases in the macrophage death, a series of nine caspase inhibitors were tested for inhibition of cell death. The caspase-2 specific inhibitor (Z-VDVAD-FMK) blocked macrophage cell death caused by RB51 or RA1 98.2 %±0.5% or 80.3%±5.2%, respectively, compared to untreated controls at 24 h post infection (Figure 6). The caspase-2 inhibitor appears to exert a greater influence in preventing cell death in RB51-infected macrophages than in RA1-infected macrophages; it might be attributed to the differences in the gene mutations of these two strains. The difference in inhibition of macrophage cell death provided by the caspase-2 inhibitor over that of the pan-caspase inhibitor may be attributed to the difference in the specificities of these inhibitors. The inhibitors of caspase -1, 3, 4, 6, 8, 9, 10, and 13 failed to significantly inhibit RB51 and RA1-induced macrophage cell death in our studies. These results support our hypothesis that the rough *Brucella abortus* strains induce macrophage cell death is mainly mediated by caspase-2.

### Caspase-2 is activated at an early stage of infection

**Figure 7:**
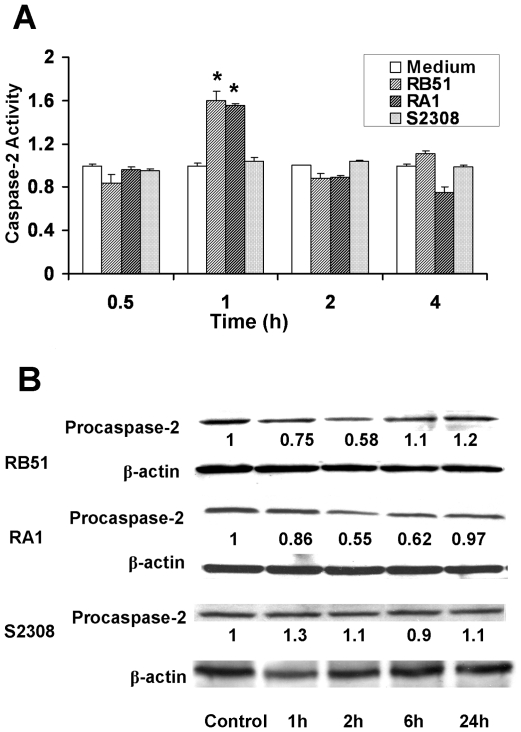
Caspase-2 enzyme activity in infected macrophages induced by RB51 but not by S2308. (A) Analysis of caspase-2 activities by a colorimetric assay. The asterisk (*) represents significant differences (*P*<0.05) compared to the medium control group. (B) Western blot analysis of precaspase-2 in infected macrophages. The numbers shown in the figure represent the amounts of procaspase-2 quantified by densitometry and normalized to the β-actin content. The data represent the means±standard deviations from three independent experiments.

Caspase-2 activation was analyzed by caspase colorimetric assay and procaspase cleavage assay. The caspase colorimetric assay showed that caspase-2 enzyme activity of RB51 and RA1 infected macrophages increased approximately 59.9%±8.3% (*p*<0.05) and 55.7%±1.9% (*p*<0.05) respectively at 1 h post infection. This increase was transient. It was followed by a rapid return to the base line at 2 h post infection (Figure 7A). Capsase-2 activation was analyzed further by detection of the cleavage of procaspase-2 protein [29]. As ascertained by western blot analysis, transient decrease in the level of procaspase-2 (48 kDa) in RB51 or RA1-infected macrophages was observed at 1 h, 2 h and 6 h post infection (Figure 7B). This confirmed that the activation of caspase-2 occurs at the early stage of infection. In contrast, S2308 did not induce significant changes in the caspase-2 enzyme activity or the level of procaspase-2 (Figure 7A and B). Caspase-1 activity was also examined in RB51 or RA1-infected macrophages. No significant changes were observed (data not shown).

The above results indicate that rough strains RB51 and RA1 induce similar apoptotic and necrotic cell death in macrophages and that the cell death is mediated by caspase-2. We later focused on comparing RB51 and its parent smooth strain 2308 to further study the apoptotic and necrotic cell death mechanism induced by rough *B. abortus*.

### Inhibition of caspase-2 activity increases RB51 survival in macrophages

**Figure 8:**
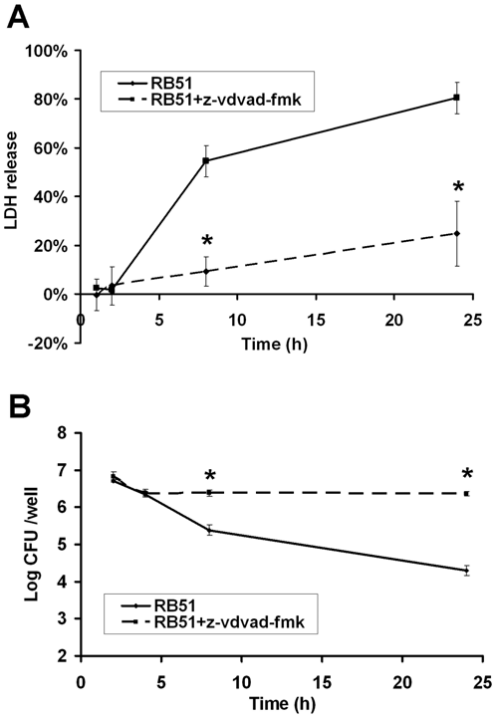
Decreased cell death of infected macrophages and increased RB51 survival inside macrophages with the usage of the caspase-2 inhibitor. Macrophages pretreated with Z-VDVAD-FMK were infected with RB51 for 24 h at a MOI of 200. (A) Macrophages pretreated with the caspase-2 inhibitor and infected with RB51 released less LDH than infected cells lacking inhibitor. (B) Growth kinetics of RB51 in macrophages. The number of RB51 cells surviving inside macrophages pretreated with the caspase-2 inhibitor was approximately 120 times greater than the number of cells found in untreated macrophages (*P*<0.05). The asterisk (*) represents significant difference (*P*<0.05) in the LDH release or bacterial CFUs from RB51-infected macrophages with caspase-2 inhibitor treatment compared to that without caspase-2 inhibitor. Data represent the means±standard deviations from three independent experiments.

To confirm the inhibition of programmed macrophage cell death after treatment with the caspase-2 inhibitor, the LDH release assay was used to monitor cytopathic cells (Figure 8A). The LDH released from RB51-infected macrophages reached a value of 54.6%±6.5% of the total LDH available in both living and dying cells at 8 h post infection, and 80.5%±6.4% at 24 h post infection. In contrast, after pretreatment with the caspase-2 inhibitor, the yield of LDH released was 9.3%±6.0% and 24.7%±13.3% at 8 h and 24 h post infection, respectively.

Inhibition of caspase-2 activity also increased the number of RB51 survived inside macrophages (Figure 8B). The inhibitor did not change the phagocytosis of RB51 by macrophage cells as demonstrated by similar number of living brucellae with or without the caspase-2 inhibition at 1 h post infection. At 8 h post infection, approximately 5% of RB51 survived inside infected macrophage cells. However, after the pretreatment of the macrophages with the caspase-2 inhibitor prior to RB51 infection, approximately 36% of brucellae survived in the macrophages. At 24 h post infection, the number of RB51 surviving inside the macrophages pretreated with the caspase-2 inhibitor was approximately 120 times greater than that inside untreated macrophages (Figure 8B). This phenomenon might be related to more cells survived due to the inhibition of macrophage cell death.

### Knockdown of caspase-2 expression decreases RB51-induced macrophage cell death

**Figure 9:**
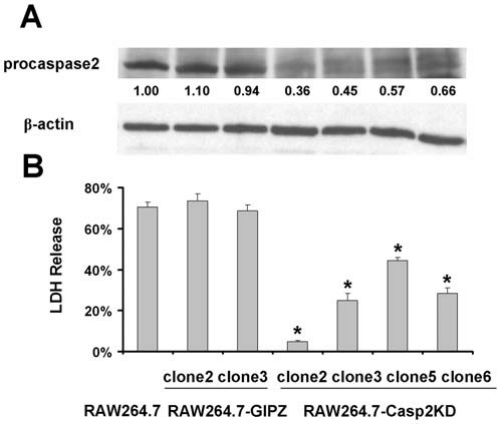
Inhibition of RB51-induced macrophage cell death by caspase-2 shRNA. Macrophages with or without caspase-2 shRNA were infected for 6 h with RB51 at a MOI of 200. (A) Caspase-2 expression in different clones was detected by Western blot analysis. The numbers shown in the figure represent the amounts of procaspase-2 that were quantified by densitometry and normalized to the β-actin data. (B) The LDH assay was used to detect differential cell death in different macrophage clones. The asterisk (*) represents significant differences (*P*<0.05) of LDH releases from different clones compared to that from the parent RAW264.7 cells. The data represent the means±standard deviations from three independent experiments.

Using different specific caspase inhibitors, the present studies demonstrate that caspase-2 plays an important role in rough *B. abortus* induced macrophage cell death. To address potential concerns as to the specificity of the caspase inhibitors [43], the RNA interference (RNAi) technique [44] was used to further identify the role of capase-2. RNAi is a natural, evolutionarily conserved regulatory mechanism that is mediated by the introduction of dsRNA into the cytoplasm of a host cell. RNAi has provided a unique tool for sequence-specific silencing to develop practical strategies for studying gene function, biological processes and pathway analysis [44]. Short hairpin RNA (shRNA) is a sequence of RNA that makes a tight hairpin turn that silences gene expression via RNAi [45]. Macrophages with specific gene knockdown were generated by transfection of macrophages with lentivirus vectors expressing gene-specific shRNA [46]. Four caspase-2 knockdown clones (RAW264.7-Casp2KD) and two control clones (RAW264.7-GipZ) were selected based on EGFP fluorescence detection. The protein expression of caspase-2 was further monitored by Western blot analysis (Figure 8A). RAW264.7-GipZ cells did not exhibit any difference in procaspase-2 expression compared with the original RAW264.7 cells. The expression levels of procaspase-2 protein in four RAW264.7-Casp2KD clones decreased by 34–64%. When infected with RB51 at a MOI of 200 for 6 h, the control RAW264.7-GipZ clones demonstrated a similar rate of cell death compared to the original RAW264.7 (68–74% vs. 71%). In contrast, the RAW264.7-Casp2KD clones showed a significantly lower rate of cell death rate (5–44%) (*P*<0.05) (Figure 9B). Of the four RAW264.7-Casp2KD clones, clone two had the lowest cell death rate (5%±0.6%) and is correlated with the lowest caspase-2 protein expression (Figure 9). These results confirm that RB51-induced apoptosis is mediated specifically by caspase-2.

### Mitochondrial membrane potential (MMP) decreases following cytochrome c release

**Figure 10:**
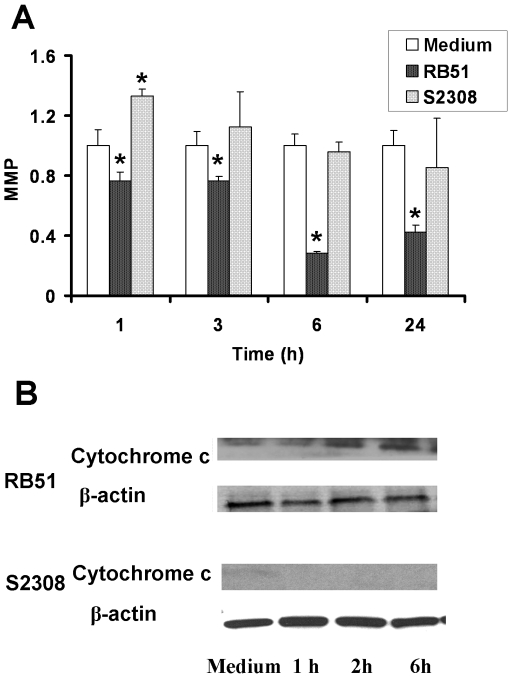
Decreased mitochondrial membrane potential (MMP) and increased cytochrome *c* release from mitochondria in RB51-infected macrophages (MOI: 200). Infection with S2308 at a MOI of 2,000 did not cause decrease of MMP or cytochrome *c* release. (A) The results of flow cytometry results were summarized in graphic form, and represent the means±standard deviations of three independent experiments. The asterisk (*) represents significant differences (*P*<0.05) compared to the medium control group. (B) Cytochrome *c* release was analyzed by Western blot analysis. β-actin was used as a reference. Only cytoplasmic portion of cell lysates was assayed. The results are a representative of three independent experiments.

Mitochondria are viewed as the major players in both necrotic and apoptotic cell death [47], [48]. A key feature of apoptotic and necrotic cascades is the initiation of the mitochondrial permeability transition (MPT) followed by the release of cytochrome *c* from mitochondria. We hypothesized that RB51 induced apoptotic macrophage cell death by activating a mitochondria-dependent apoptotic pathway. Increased MPT results from a decrease of MMP. Decreased MMP promotes a decrease in the retention of the lipophilic cationic dye 3,3′-dihexyloxycarbocyanine iodide (DiOC6). This phenomenon is frequently used to quantify MMP through flow cytometry [49] and was applied in this study. A MMP decrease of 23.5%±5.8% (*P*<0.05) was detected in RB51-infected macrophages at 1 h post infection. At 6 h post infection, the level of MMP decreased to 28.5%±0.7% (*P*<0.05) of the value of uninfected macrophages. The MMP of RB51-infected macrophages remained at a low level for 24 h post infection (Figure 10A). The MMP of S2308-infected macrophages increased 33.0%±10.5% (*P*<0.05) at 1 h post infection but after 2 h post infection returned to the level found with the uninfected macrophages (Figure 10A).

A direct effect of the increased MPT and decreased MMP is the release of cytochrome *c* from the mitochondria to the cytoplasm. To test this effect, the cytochrome *c* levels from the cytosol of infected and uninfected macrophages from the same experiment were analyzed (Figure 10B). Release of cytochrome *c* into the cytoplasm of RB51-infected macrophage was detectable as early as 2 h post infection (Figure 10B). No cytochrome *c* was detected in the cytoplasm of S2308-infected macrophage before 24 h post infection.

## Discussion

The results reported here indicated that attenuated rough *B. abortus* strains RB51 or RA1, but not their parent smooth virulent strain 2308, induced both apoptosis and necrosis in RAW264.7 macrophage cells, bone marrow-derived macrophages, and peritoneal macrophages. The programmed cell death induced by rough *B. abortus* is mediated primarily by caspase-2 and through a mitochondria-dependent cell death pathway. Since rough strains RB51 and RA1 have deficient LPS O antigen found in their parent strain S2308, the rough *Brucella* phenotype with deficient O antigen plays a critical role in caspase-2-mediated macrophage cell death induced by rough *Brucella*.

Cell death of macrophages infected with rough *Brucella* strains has been discovered by several groups [9], [10], [11], [12], [13]. The discovery of cell death induced by rough *Brucella* was first reported by Freeman *et al* in 1961 [11]. Fernandez-Prada *et al* later found that a rough *B. melitensis* mutant WRR51 lacking a *wboA* gene carried by smooth virulent *B. melitensis* strain 16 M resulted in apoptotic cell death in human monocytes from healthy volunteers [9]. Our results are consistent with this finding. Pei *et al* also showed that several rough *B. abortus* strains obtained from a transposon mutation induced necrotic and oncotic macrophage cell death [12]. No apoptotic macrophage cell death was observed in this report. One possible reason for the difference in the observed cell death patterns might be that different rough mutants were used in each study [12], [50]. The rough phenotype can result from mutations affecting O antigen precursor synthesis, its polymerization and transport, or from defects in the inner core oligosaccharide [50]. The structure of rough lipopolysaccharide present in each mutant may vary. Different levels of necrotic/oncotic macrophage cell death may also be induced by rough *B. abortus* mutants with distinct gene mutations. For example, at the MOI of 200, macrophage cell death was induced by rough strain CA180 (mutation of *manA* encoding phosphomannomutase) but not by rough strain CA533 (mutation of a *rfbD*-like gene encoding a putative GDP-D-mannose dehydratase) at 8 h post infection [12]. The observation that RB51 and RA1 induce similar patterns of macrophage cell death suggests that the *wboA* gene mutation in RB51 is a primary player in the apoptotic and necrotic cell death.

The absence of *Brucella* O antigen may not directly contribute to the induction of macrophage cell death by rough *Brucella*. While RB51 does not induce detectable antibodies in animals against *Brucella* O antigen, RB51 has been found to produce a low but detectable level of O antigen [51]. Gonzalez et al. [50] generated rough *Brucella* mutants by transposon mutagenesis of more than 10 genes involved in O antigen (or O chain) or the core oligosaccharide biosynthetic pathways. These rough mutants are classified into three types based on the decrease in LPS molecular weight. Therefore, rough *Brucella* strains are defined by deficient O antigen instead of completely deleted O antigen. The reason of macrophage cell death induced by rough *Brucella* does not rely on the complete deletion of O antigen. Instead, the cell death is most likely due to the pleiotropic effect of removing the majority of the O antigen that results in the change of the *Brucella* cell envelope structure. The LPS defects alter key topological, physiochemical and biological surface properties of rough *Brucella* [50]. The outer membrane proteins and lipids in rough *Brucella* cells are readily exposed to the host environment, making them much easier to interact with the host cells. It has also been found that the surface of rough *Brucella* out membrane is highly hydrophobic and negatively charged [50]. Such a surface may allow multiple nonspecific interactions with eukaryotic membranes. Therefore, *Brucella* O antigen probably acts as a negative modulator of non specific adherence which allows receptors to function [50]. It has been observed that rough mutants penetrate more actively and display higher adherence to macrophages than smooth strains [52], [53], [54]. Rough and smooth *Brucella* strains enter macrophages through different mechanisms. Virulent smooth *Brucella* cells use lipid rafts to enter murine macrophages while rough *Brucella* cells do not [55]. We found similar numbers of internalized brucellae inside macrophages when rough strain MOI was 200 and smooth strain MOI was 2000 (Figure 1). The MOI of 200 of rough strain induced significant cell death, while smooth strain 2308 did not induce obvious cell death even with an MOI of 2000. The results also suggest that different outcomes of macrophage cell death is primarily due to the difference of rough vs. smooth phenotypes instead of the different numbers of phagocytosed *Brucella* cells.

Pei et al [9], [10], [11], [12], [13] found that viable intracellular rough *Brucella* were required for inducing cytopathic effect on infected macrophages. Although heat-killed rough brucellae were internalized as efficiently as live bacteria, they did not induce macrophage cell death [9], [10], [11], [12], [13]. Our study is consistent with the conclusion although a very high MOI of 2,000 induced limited but detectable macrophage cell death at late infection stage (Table 1). Pei et al. [12] further found that protein synthesis from live rough *Brucella* cells is required for induction of macrophage cell death. It was shown that inhibition of bacterial synthesis and replication by chloramphenicol treat prevented cell death of infected macrophage [13]. It is interesting that viable but not killed smooth *Brucella* inhibit spontaneously occurring apoptotis in human macrophages [5], [6], [7]. How live rough *Brucella* promotes apoptotis and live smooth *Brucella* inhibits apoptosis is an important question for better understanding *Brucella* pathogenesis and host responses against *Brucella* infections.

The relationship between rough *Brucella*-induced apoptotic and necrotic macrophage cell death may be complex. Necrosis refers to the sum of changes secondary to cell death by another mechanism such as apoptosis [17], [21], [22]. Our study showed that macrophage cell death induced by rough *Brucella* was dose-dependent since more cell death was observed with higher MOIs (Table 1, Figure 3). Meanwhile, the numbers of internalized bacteria in infected macrophages might not be even. Some macrophages phagocytosed more rough bacteria than others with the same MOI (Figure 3). Macrophages infected with more rough *Brucella* cells tended to induce more and quicker macrophage cell death. Necrosis occurred more than apoptotis in the late stage of *Brucella*-induced macrophage cell death. Therefore, it is likely that apoptotic and necrotic cell death of *Brucella*-infected macrophages represents different stages of the same cell death pathway, from early and late apoptosis to eventual necrosis. Alternatively, a non-apoptotic pathway(s) of cell death might be induced in some infected macrophages, which results in necrosis. Further investigation is needed to test this possibility.

Caspase-2 is activated in response to embryonic development [56] and many biological stresses, including DNA damage, aging, UV irradiation, tropic factor withdrawal, endoplasmic reticulum stress, death receptor activation and heat shock [57], [58], [59]. Deficient caspase-2 activation has been observed in gastric cancer, and acute myeloblastic leukemias [60], [61]. Caspase-2 activation has also been observed in host cells infected with *Salmonella* [29] and Epstein-Barr virus [62]. *Salmonella typhimurium* induced primarily caspase-1-mediated pro-inflammatory pyroptosis [23], [24], [25]. The action of caspase-2 was also needed for *Salmonella*-induced apoptosis as shown by the results of experiments using caspase-2 knockout macrophages and chemical caspase-2 inhibitors [29]. Unlike with *S. typhimurium*, rough (but not smooth) *B. abortus*-induced macrophage cell death exhibits caspase-2 activation and is noticeable hampered by a caspase-2 inhibitor and caspase-2-specific shRNA (Figure 6 and 9). On the other hand, the macrophage cell death induced by rough *B. abortus* strains RB51 and RA1 were not obviously inhibited by a caspase-1 inhibitor (Figure 6). These findings suggest that caspase-1 plays minimal role in the *Brucella*-induced cell death. Loss of mitochondrial integrity and release of cytochrome *c* do not occur during pyroptosis [17] but was observed in macrophages infected with RB51 and RA1. This difference indicates that pyroptosis does not play a major role in rough *Brucella*-induced cell death. Unlike virulent *Salmonella* and *Shigella* strains which induce apoptosis in infected macrophages, smooth virulent *Brucella* strains inhibit programmed macrophage cell death and replicate in macrophages [5], [6], [7], [9], [63]. The current study further indicates that *B. abortus* smooth strain S2308 does not induce caspase-2 activation or macrophage death. Therefore, the caspase-2-mediated macrophage apoptosis induced by rough attenuated strains but inhibited by the parent wild type appears to be a novel findings not as yet found in other bacterial pathogen.

How caspase-2 mediates programmed cell death of macrophages infected by rough *Brucella* is unclear and deserves further investigations. Across species caspase-2 is one of the most conserved caspases. Its function in cell death signaling is still an enigma [64]. Caspase-2 is unique among the caspases in that it has features of both upstream and downstream caspases. Caspase-2 contains a long CARD prodomain that can be used to interact with adaptor proteins. This is typical of initiator caspases. On the other hand, the predicted cleavage specificity of caspase-2 appears to place it to be an effector caspase similar to caspases-3 and -7 [58]. The present studies reveals that caspase-3 or caspase-8 inhibitor slightly yet not significantly inhibits RB51- and RA1-induced macrophage cell death. The caspase-3 inhibitor (Z-DEVD-FMK) was reported to also inhibit caspase-7 activity [65]. It is possible that caspase-2 functions as an initiator caspase and regulates apoptotic activities of other effector caspases (e.g., caspase-3, -7 and -8). It is also possible that caspase-2 regulates *Brucella*-induced cell death directly by acting as an effector caspase. This hypothesis is supported by the observation that caspase-3 and caspase-8 have the potential to regulate downstream caspase-2 activity [7], [64], [66]. An alternative possibility is that caspase-2 may function as an initiator caspase and effector caspase at the same time.

The study provides new evidence to support the hypothesis that smooth virulent *Brucella* has the ability to control or influence mitochondrial functions, especially those related to programmed cell death in macrophage cells, via a mechanism that is a crucial survival mechanism in this pathogen. Virulent *Brucella* strain S2308 appears to inhibit macrophage cell death by inhibiting mitochondrial membrane transition and cytochrome *c* release (Figure 10). The inhibition of macrophage cell death allows *Brucella* survive and replicate inside macrophages. In contrast, rough *Brucella* strains induce macrophage cell death through the release of cytochrome *c* from the mitochondria. The dying macrophages release *Brucella* to a more hostile environment outside macrophages. It is interesting to note that the *Brucella* species are classified as α-Proteobacteria, the modern-day relatives of an ancient endosymbiont that gave rise to mitochondria [67], [68]. Our previous study indicates virulent *B. melitensis* strain 16 M inhibited mitochondria activities at an early stage of infection [6]. The interactions between mitochondrial permeability transition and caspase-2 activation in the apoptotic cell death are still unclear. It has been repeatedly proposed that caspase-2 can act upstream of mitochondrial events as an initiator that triggers cytochrome *c* release and subsequent apoptosome formation, either directly or through generation of tBid [58], [64], [69]. Other studies place caspase-2 neither upstream nor downstream but suggest it acts in concert with the mitochondrial as well as the death receptor pathways of apoptosis [70]. Our results suggest that programmed cell death in macrophages infected with rough *B. abortus* is at least partially due to the mitochondrial permeability transition and cytochrome *c* release. The relationship between mitochondrial permeability transition and caspase-2 activation in the apoptotic cell death of *Brucella*-infected macrophages requires further investigation.

The caspase-2-mediated cell death of macrophages infected with vaccine strain RB51 and vaccine candidate RA1 may help us to better understand the process of protective *Brucella* immunity. Programmed cell death of macrophages infected with many pathogens (e.g., *M. tuberculosis*, *Salmonella*, Influenza virus) plays an important role in antigen presentation to T cells through a process called cross-priming [71]. It is likely that rough *Brucella*-induced macrophage cell death may play an important role in protective immunity against brucellosis through cross-priming or other mechanisms. Further understanding of *Brucella*-induced programmed cell death will undoubtedly help decipher *Brucella* pathogenesis and promote rationale *Brucella* vaccine development.

## Materials and Methods

### Bacterial strains and macrophage cell culture

*B. abortus* vaccine strain RB51, strain RA1 and the parent wild type strain S2308 were originally obtained from Dr. Gerhardt G. Schurig's laboratory at Virginia Tech. *B. abortus* strain RB51GFP that contains a GFP plasmid (pNSGroE/GFP) was a gift from Dr. Nammalwar Sriranganathan's laboratory at Virginia Tech [37]. These bacterial strains were grown either in tryptic soy broth or on tryptic soy agar plates. Heat-killed *Brucella* was prepared by boiling bacteria for 20 min. The characteristics of these strains were confirmed with AMOS (*abortus-melitensis-ovis-suis*) PCR reactions [72], crystal violet staining, antibiotic resistance, urease test, and thionin and fuchsin susceptibility [73]. To minimize naturally mutated rough phenotypes in smooth strain S2308, one single colony of smooth phenotype S2308 was selected based on crystal violet staining and used for bacterial amplification. All studies utilizing *Brucella* strains were performed in a Biosafety level 3 (BSL-3) laboratory. Murine macrophage cell line RAW264.7 (ATCC #TIB-71) was cultured at 37°C with 5% CO_2_ in complete tissue culture medium (c-DMEM) consisting of Dulbecco's modified Eagle's medium (DMEM; ATCC) supplemented with 10% heat-inactivated fetal bovine serum (HyClone, Logan, Utah). Macrophages between passages 4 through 15 were used in these experiments. Bone marrow-derived macrophages (BMDM) were prepared from BALB/C mice and were cultured at 37°C with 5% CO_2_ in RPMI-1640 supplemented with 10% heat-inactivated fetal serum and 30% L-Cell Medium as previously described [74]. Bacterial and macrophage cells were maintained under conditions that sustained exponential growth.

### Macrophage infection assays

Macrophage cells were cultured in 96-well, 24-well, or six-well plates. In the macrophage infection experiments, 5×10^4^ cells per well were seeded in 96-well plate and 2.5×10^5^ cells per well were seeded in 24-well plate. For flow cytometry assays, 1×10^6^ cells were seeded per well in six-well plate. The macrophages were cultured overnight prior to infection with RB51, RA1 or S2308 cells. Macrophages were infected with *Brucella* at a multiplicity of infection (MOI) of 20, 200 or 2,000. The plates were centrifuged at 300 x *g* for 5 min. All incubations with *Brucella* were conducted at 37°C in an atmosphere containing 5% (vol/vol) CO_2_. After 30 min the cells were washed three times with Dubecco's phosphate buffered saline (DPBS), and incubated in fresh DMEM supplemented with 50 µg/ml of gentamicin to kill extracellular bacteria. A specific caspase inhibitor might be included in the cell culturing. For caspase inhibition studies, a caspase inhibitor sample pack (R&D systems Inc, Minneapolis, US) was used which contains a pancaspase inhibitor (Z-VAD-FMK) and nine specific caspase inhibitors including those for inhibition of caspase-1 (Z-WEHD-FMK), caspase-2 (Z-VDVAD-FMK), caspase-3 (Z-DEVD-FMK), caspase-4 (Z-YVAD-FMK), caspase-6 (Z-VEID-FMK), caspase-8 (Z-IETD-FMK), caspase-9 (Z-LEHD-FMK), caspase-10 (Z-AEVD-FMK), and caspase-13 (Z-LEED-FMK). The macrophages were pretreated with the individual caspase inhibitors at a final concentration of 20 µM for 1 h prior to infection.

### Determination of programmed macrophage cell death

Macrophages were infected in the presence, or absence, of a caspase inhibitor as described above. Apoptotic or necrotic macrophages were detected using two approaches. In the first approach, cells were stained with Annexin V (green dye) and propidium iodide (PI, red dye) using an Annexin V-FLUOS staining kit (Roche Diagnostics Corporation, Indianapolis, Ind.). RB51, RA1 or S2308-infected macrophages were incubated with Annexin V and PI at room temperature for 20 min and observed by fluorescence microscopy (Nikon TE2000-S microscope). Images were photographed with an RT Slide Spot digital camera. Apoptotic and necrotic cell numbers were counted in representative fields containing at least 200 cells. In the second approach, the nuclei of RB51 infected cells and untreated control cells were stained with the blue-fluorescent Hoechest 33342 (Invitrogen, OR)at 1 µg/ml in DPBS at room temperature for 15 min and observed by fluorescence microscopy. Gliotoxin-treated cells were employed as a positive control for apoptotic cell death, and *t*-butyl-hydroperoxide (TBH)-treated cells served as a positive control for necrotic cell death [12], [13].

### Quantitation of cell viability

Cells were cultured in triplicate in 96-well plates infected with RB51, RA1 or S2308 as described above. The culture supernatants were collected at various time points, and the lactate dehydrogenase (LDH) released determined by a CytoTox 96 nonradioactive cytotoxicity assay (Promega, Madison, Wis.) according to the manufacturer's instructions. To reduce the LDH background from fetal bovine serum, the supernatants were diluted 1∶1 with phosphate buffered saline (PBS) before the assay. The percent of membrane-damaged dying cells is expressed as a percentage of maximum LDH release, i.e., 100× (optical density at 490 nm [OD_490_] of infected cells - OD_490_ of uninfected cells)/(OD_490_ of lysed uninfected cells - OD_490_ of uninfected cells). The data presented represent the average±standard deviation of at least three separate experiments.

### Assay of *Brucella* survival inside macrophages

Macrophages were seeded in a 24-well plate with 2.5×10^5^ cells per well and incubated overnight as described above. RB51, RA1 and 2308 were used to infect macrophages with varying MOIs (see above). To assess intracellular survival of *Brucella* inside macrophages, the cells were lysed with 1 ml 0.1% (vol/vol) Triton X-100 in sterile water at selected time points (1, 6 and 24 h). The colony forming units (CFUs) were obtained by plating a series of dilutions on TSA plates [75]. All experiments were conducted in triplicate.

### Western blot analysis

Cells cultured in six-well plates were infected with RB51, RA1 or S2308 as described above. Cell lysis of the cytosolic fraction was prepared with a Mitochondria/Cytosol Fractionation Kit (Biovision) according to the manufacturer's instructions. Lysates (50 µg per sample) were analyzed by Laemmli SDS-PAGE and transferred onto Immobilon P membranes (Millipore) at 150 V for 1 h in the presence of transfer buffer (25 mM Tris, 192 mM glycine, 15% methanol, pH 8.3). After transfer, the membranes were blocked for 1 h a blocking solution (5% low fat dried milk dissolved in TBS-T (25 mM Tris-HCl, 150 mM NaCl, 0.5% Tween 20, pH 7.4) and were probed overnight at 4°C with the mouse anti-caspase-2 (Cell signaling, CA), Rabbit anti-β-actin (Biovision, CA) or mouse anti-cytochrome *c* (NeoMarkers, CA) antibodies. Membranes were washed with PBS-T and incubated with HRP conjugated to either goat anti-rabbit IgG or goat anti-mouse IgG (Biovision, CA) at room temperature for 1 h. After extensive washing with TBS-T, the antigen was visualized using an ECL Western Blotting Substrate (Pierce). The results were quantified by densitometry and normalized to the β-actin data. This experiment was conducted in triplicate.

### Caspase-1 and -2 activity assays

Macrophages were cultured in T-25 flasks infected with RB51 or S2308. The caspase-1 and -2 activities were determined by monitoring proteolysis of the appropriate colorimetric substrates using the Caspase Colorimetric Assays (R&D Systems, CA). WEHD and VDVAD served as substrates employed to detect caspase-1 and -2 enzyme activities, respectively. All experiments were conducted in triplicate. The whole-cell lysate was added to a buffer containing 200 μM of the appropriate caspase-specific substrate conjugated to *p*-nitroanilide. After an incubation of 37°C for 3 h, caspase activity was quantified spectrophotometrically at a wavelength of 405 nm using a VERSAmax microplate reader (Molecular Devices).

### Preparation of stable caspase-2 knockdown macrophage cell lines

Lentivirus vectors Lenti-Casp2miRNA-VSVG (Open Biosystems OligoID V2LMM_16681) used for producing anti-caspase-2 shRNA and empty lentivirus vector Lenti-GipZ-VSVG were generated at the University of Michigan shRNA Core Facility. For transduction, RAW264.7 cells were planted into six-well tissue culture plates at 2.5×10^5^ per well. On the following day, the cell medium was aspirated off and replaced with 1.25 ml viral supernatant (MOI of 10) containing 8 µg/ml polybrene into each well. The plates were gently rocked and centrifuged at 2,500 rpm for 90 min at 30°C using an Eppendorf 5810R centrifuge. The medium was replaced after 24 h, and the cells were monitored for enhanced green fluorescent protein (EGFP) expression. To select the stably transfected cells, at 48 h after transfection, cells were scraped down and planted to 10 cm cell culture dish, selected with c-DMEM containing 10 µg/ml puromycin. Stably transfected cells were selected with the presence of puromycin for over 4 weeks. Puromycin-resistant clones were picked and expanded. The caspase-2 expression of each clone was examined by Western blot analysis.

### Analysis of mitochondrial membrane potential (MMP)

The loss of mitochondrial membrane potential (ΔΨ_m_) results in a decrease in the retention of 3,3′-dihexyloxycarbocyanine iodide (DiOC_6_). The ΔΨ_m_ was evaluated by measuring the retention of the lipophilic cationic dye DiOC_6_ in mitochondria [49]. Briefly, cells cultured in six-well plates were infected with RB51 or S2308 in triplicate as described above. At various time points post infection, the cells were preloaded with 1.5 nM DiOC_6_ in c-DMEM incubated at 37°C for 20 min, washed and fixed with 1% paraformaldehyde at 25°C for 20 min. The cells were then dislodged with a rubber policeman, pelleted at 500 x *g*, washed, and resuspended in PBS containing 1% paraformaldehyde. Flow cytometry to detect cells with diminished fluorescence was performed under FL-1 logarithmic amplification using a FACSCanto Flow Cytometer (BD Bioscience). Macrophages stained with an irrelevant isotype antibody source served as controls.

### Analysis of cell death of peritoneal primary macrophage vaccinated with RB51

Female BALB/c mice (three per treatment group) were injected intraperitoneally with 0.2 ml of a 0.15 M NaCl saline solution (controls) or 0.2 ml of saline containing approximately 5×10^8^ CFUs of strain RB51, heat-killed RB51 (HK-RB51), or S2308. This dose was routinely used for RB51 vaccine efficacy studies [39], [40]. At 2 h post infection, peritoneal macrophages were isolated from both RB51-immunized and saline-injected mice by washing the cavity with cold PBS. Cell suspensions were washed twice with cold complete tissue culture medium (RPMI 1640 with 10% fetal calf serum and 50 µg/ml gentamicin). Isolated peritoneal cells were counted with 0.4% trypan blue and plated in complete tissue culture medium at a concentration of 1×10^6^ cells per well in a six-well plate. After incubation for 60 min at 37°C in a 5% CO_2_ atmosphere, the nonadherent cells were removed by washing them five-time in 2 ml PBS hold at 37°C. The peritoneal macrophages were incubated in fresh RPMI 1640 supplemented with 50 µg/ml of gentamicin. Annexin V-FLUOS staining kit (Roche Diagnostics Corporation, Indianapolis, Ind.) was used to detect the dead peritoneal macrophage cells 24 h post infection.

### Statistical data analysis

Statistical significance was determined using Student's *t*-test for data with two groups and one-way analysis of variance for multiple group comparison; a *P* value of <0.05 was considered significant.
